# Investigation of the anti-TB potential of selected propolis constituents using a molecular docking approach

**DOI:** 10.1038/s41598-018-30209-y

**Published:** 2018-08-16

**Authors:** Mohammad Tuhin Ali, Natalia Blicharska, Jamil A. Shilpi, Veronique Seidel

**Affiliations:** 1 Department of Biochemistry and Molecular Biology, University of Dhaka, Dhaka, Bangladesh; 2 Victoria College, University of Toronto, Toronto, Canada; 3 Pharmacy Discipline, Life Science School, Khulna University, Khulna, Bangladesh; 4 Natural Products Drug Discovery Research Group, Strathclyde Institute of Pharmacy and Biomedical Sciences, University of Strathclyde, Glasgow, UK; 5 Present Address: Department of Pharmaceutical and Biological Chemistry, UCL School of Pharmacy, London, UK

## Abstract

Human tuberculosis (TB), caused by *Mycobacterium tuberculosis*, is the leading bacterial killer disease worldwide and new anti-TB drugs are urgently needed. Natural remedies have long played an important role in medicine and continue to provide some inspiring templates for drug design. Propolis, a substance naturally-produced by bees upon collection of plant resins, is used in folk medicine for its beneficial anti-TB activity. In this study, we used a molecular docking approach to investigate the interactions between selected propolis constituents and four ‘druggable’ proteins involved in vital physiological functions in *M*. *tuberculosis*, namely *Mt*PanK, *Mt*DprE1, *Mt*PknB and *Mt*KasA. The docking score for ligands towards each protein was calculated to estimate the binding free energy, with the best docking score (lowest energy value) indicating the highest predicted ligand/protein affinity. Specific interactions were also explored to understand the nature of intermolecular bonds between the most active ligands and the protein binding site residues. The lignan (+)-sesamin displayed the best docking score towards *Mt*DprE1 (−10.7 kcal/mol) while the prenylated flavonoid isonymphaeol D docked strongly with *Mt*KasA (−9.7 kcal/mol). Both compounds showed docking scores superior to the control inhibitors and represent potentially interesting scaffolds for further *in vitro* biological evaluation and anti-TB drug design.

## Introduction

Human tuberculosis (TB), caused by *Mycobacterium tuberculosis*, is the leading cause of deaths worldwide from a single infectious agent. In 2016, it was estimated that 10.4 million people developed active TB disease and that this resulted in 1.7 million deaths. TB rates are particularly high in developing countries where, with HIV/AIDS and malaria, it creates a huge burden on healthcare systems. Treating TB is a long process that involves complex drug regimens, with adverse effects and interactions, and is associated with poor patient compliance. This has led to the evolution of multidrug-resistant (MDR-TB) and extensively drug-resistant (XDR) strains. The treatment of MDR-TB requires expensive drugs and XDR-TB is often incurable. The rise in resistant TB over the past decade is now a worldwide emergency^1^. Although drug development efforts have intensified in recent years, with two new anti-TB drugs (bedaquiline and delamanid) licensed and a few others currently undergoing clinical evaluations, the current drug development pipeline is still insufficient to address such a global health challenge. There remains an urgent need to discover and develop new anti-TB drugs, particularly to target drug-resistant and dormant strains of *M*. *tuberculosis* as well as providing a more effective and shorter duration of treatment^2^.

Natural remedies, sourced from plants, microbes and animal products, have for centuries played an important role in medicine. They represent a unique pool of highly-diverse chemicals that have evolved to specifically interact with biological targets and that continue to provide some new and inspiring templates for pharmaceutical drug design^3^. In recent years, there has been a renewed interest in the investigation of natural sources for the identification of novel antitubercular agents^4–8^. Propolis, also known as bee glue, is a natural substance produced by honeybees mainly upon collection of plant secretions, such as resins and sticky exudates on leaf buds and plant wounds. The word propolis is derived from Greek, in which pro means “at the entrance to” and polis means “community” or “city”. Bees use propolis as a construction and repair material to seal gaps, smooth out internal walls in their hives and as an antiseptic coating to generally protect from external contamination. Propolis has a highly variable chemical composition depending on the geographical location from where it is collected. For instance, propolis from temperate regions of the world is rich in phenolic compounds derived from poplar tree exudates whereas bees in tropical countries have different plant sources at their disposal resulting in propolis types rich in other phytochemicals such as prenylated flavonoids and benzophenones, lignans, terpenoids and phenolic lipids^9–13^. Propolis has a long history of use as a folk remedy to treat a variety of ailments^14^. Numerous scientific studies have been carried out to investigate its medicinal properties, including anti-inflammatory^15^, immunostimulant^16^, anti-oxidant^17^, antitumour^18^, neuroprotective^19^ and antimicrobial activity^12,20,21^. Interestingly, propolis has been used as an ingredient in traditional cures for tuberculosis^22–25^. Previous *in vitro* studies have demonstrated that extracts of propolis could inhibit the growth of *M*. *tuberculosis* as well as synergise the effect of established antitubercular drugs such as isoniazid, rifampicin and streptomycin^26,27^. It has also been observed that propolis inhibited the development of TB by lowering necrosis formation in granulomas of *M*. *tuberculosis*-infected animals^28^.

Several enzymes involved in vital physiological functions in *M*. *tuberculosis* have been identified as novel attractive molecular targets for anti-TB drug development^29–32^. Here, we used a guided docking approach with AutoDock Vina to predict the interactions between selected propolis constituents and four of these essential mycobacterial enzymes, namely pantothenate kinase (*Mt*PanK, type 1)^33^, decaprenylphosphoryl-β-D-ribose 2′-epimerase 1 (*Mt*DprE1)^34^, protein kinase B (*Mt*PknB)^35^ and β-ketoacyl acyl carrier protein synthase I (*Mt*KasA)^36^. Molecular docking is a popular tool used in the virtual screening of small molecules (ligands) against proteins (targets) and several studies have successfully used AutoDock Vina to investigate the interactions of natural products against specific protein targets, including mycobacterial enzymes^37–41^. The docking of propolis constituents towards *Mt*PanK, *Mt*DprE1, *Mt*PknB and *Mt*KasA, however, has never been reported.

## Results

The propolis constituents investigated in this study represent some structurally diverse compounds that we grouped into four main categories, namely flavonoids, terpenoids, simple phenolics and miscellaneous substances including a pterocarpan, a phenylethanoid derivative, five stilbenes and four lignans. Known molecules, that had been reported previously in the literature as inhibitors of the target enzymes and for which the nature and role of the binding site residues were known from their available complexes with the proteins, were used as controls. In order to validate the docking conditions prior to virtually screening the propolis constituents, each control inhibitor was retrieved from its co-crystallised complex and re-docked using the AutoDock Vina software against the relevant target. Then, a docking score for each propolis compound was calculated to estimate its binding free energy towards *Mt*PanK, *Mt*DprE1, *Mt*PknB and *Mt*KasA (Table S1). The docking score values obtained for compounds within each phytochemical class were compared to the scores of the control inhibitors for each target in order to select molecules with the lowest energy values that ranked higher than the chosen control inhibitors against the target proteins. We observed that none of the propolis constituents exhibited scores that ranked better than the controls neither against *Mt*PanK nor *Mt*PknB. Instead, only docking to *Mt*KasA and *Mt*DprE1 gave useful scores. Thus, the prenylated flavanones isonymphaeol D, isonymphaeol C and isonymphaeol B showed strong docking scores towards *Mt*KasA (−9.7, −9.6 and −9.5 kcal/mol, respectively) superior to the control inhibitor thiolactomycin (−7.9 kcal/mol). The Ki of isonymphaeol D for *Mt*KasA was estimated at 0.07 μM (control was 1.62 μM). Isonymphaeol D also showed a strong predicted binding towards *Mt*DprE1 (−10.1 kcal/mol) compared with the control inhibitor 0T4 (−9.2 kcal/mol). Among the terpenoids, we observed that the oleanane-type triterpene β-amyrin acetate showed some affinity for *Mt*DprE1 (−9.9 kcal/mol) and ranked better than 0T4 (−9.2 kcal/mol). In the simple phenolics group, (+)-chicoric acid exhibited a strong binding score (−9.5 kcal/mol) for *Mt*KasA compared with thiolactomycin (−7.9 kcal/mol). The stilbene 5-((*E*)-3,5-dihydroxystyryl)-3-((*E*)-3,7-dimethylocta-2,6-dien-1-yl) benzene-1,2-diol showed a docking score against *Mt*KasA (-9.4 kcal/mol) that also ranked better than thiolactomycin while the lignan (+)-sesamin docked strongly to *Mt*DprE1 with a score (−10.7 kcal/mol) and predicted Ki (0.01 μM) better than 0T4 (−9.2 kcal/mol and Ki of 0.18 μM) (Table 1).

**Table 1:** Predicted binding affinity (docking scores in kcal/mol) and inhibition constant (Ki in μM) of selected propolis constituents and re-docked control inhibitors against *Mt*DprE1 and *Mt*KasA^a^.

	*Mt*DprE1		*Mt*KasA	
	Docking score	Ki	Docking score	Ki
Control inhibitor, 0T4	−9.2	0.18		
Control inhibitor, thiolactomycin			−7.9	1.62
Isonymphaeol-C	−9.5	0.11	−9.6	0.09
Isonymphaeol D	−10.1	0.04	**−9**.**7**	0.07
Isonymphaeol B	−9.5	0.11	−9.5	0.11
β-Amyrin acetate	−9.9	0.05	−2	>1.10^4^
(+)-Chicoric acid	−9.1	0.21	−9.5	0.11
5-((*E*)-3,5-dihydroxystyryl)-3-((*E*)-3,7-dimethylocta-2,6-dien-1-yl) benzene-1,2-diol	−9.2	0.18	−9.4	0.13
(+)-Sesamin	**−10**.**7**	0.01	−8.7	0.42

^a^Compounds within each phytochemical class showing the lowest energy values and ranking better than any of the given control inhibitors are highlighted in bold.

**Figure 1:**
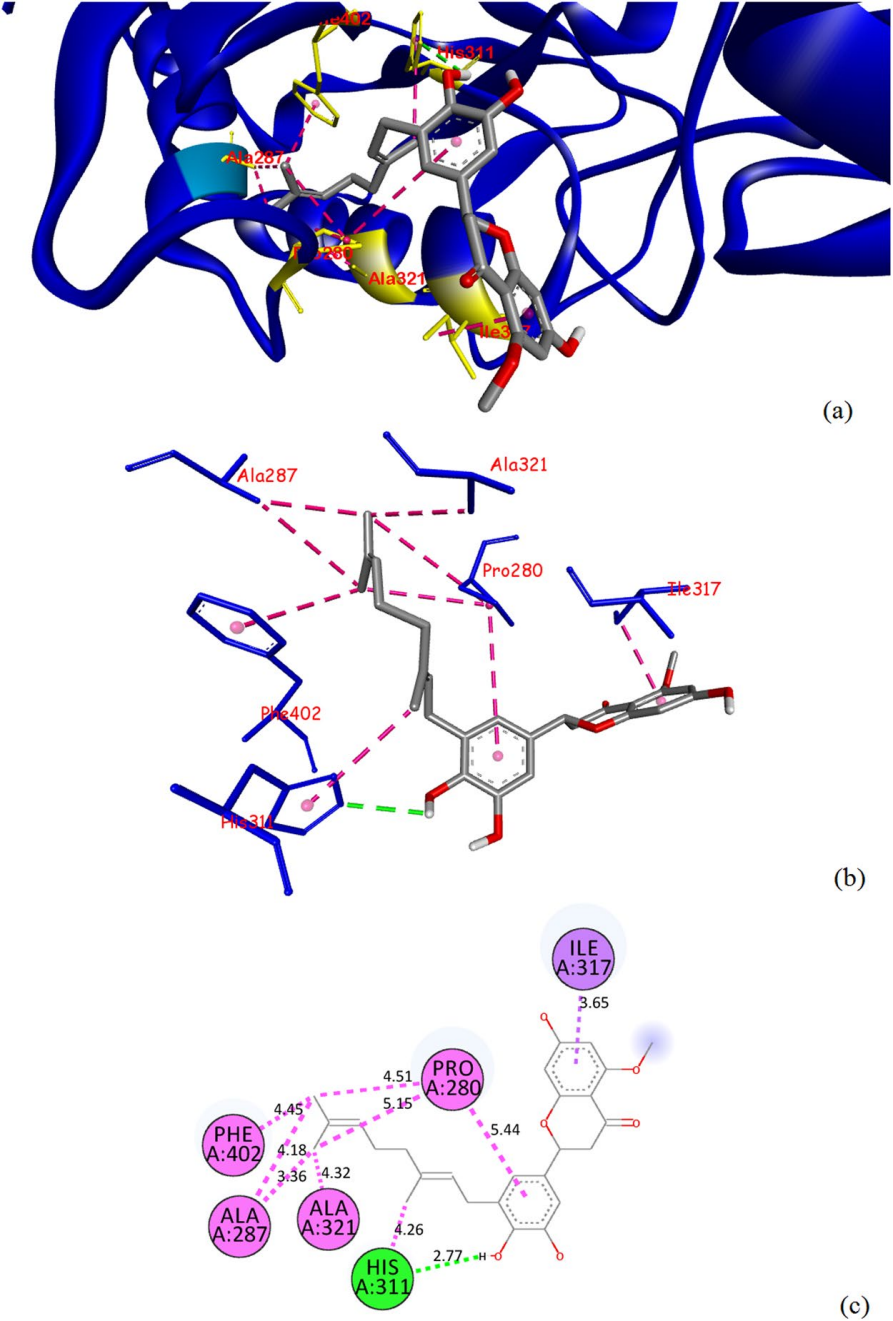
Molecular interactions between isonymphaeol D and *Mt*KasA. Docked pose of isonymphaeol D in the *Mt*KasA binding site **(a)**, interactions between isonymphaeol D and *Mt*KasA showing key hydrogen-bonds (green dashed lines), hydrophobic bonds (dark pink dashed lines) and respective amino acid residues **(b)**, 2D plot of interactions between isonymphaeol D and key residues of *Mt*KasA **(c)** generated by BIOVIA Discovery Studio visualizer.

**Figure 2:**
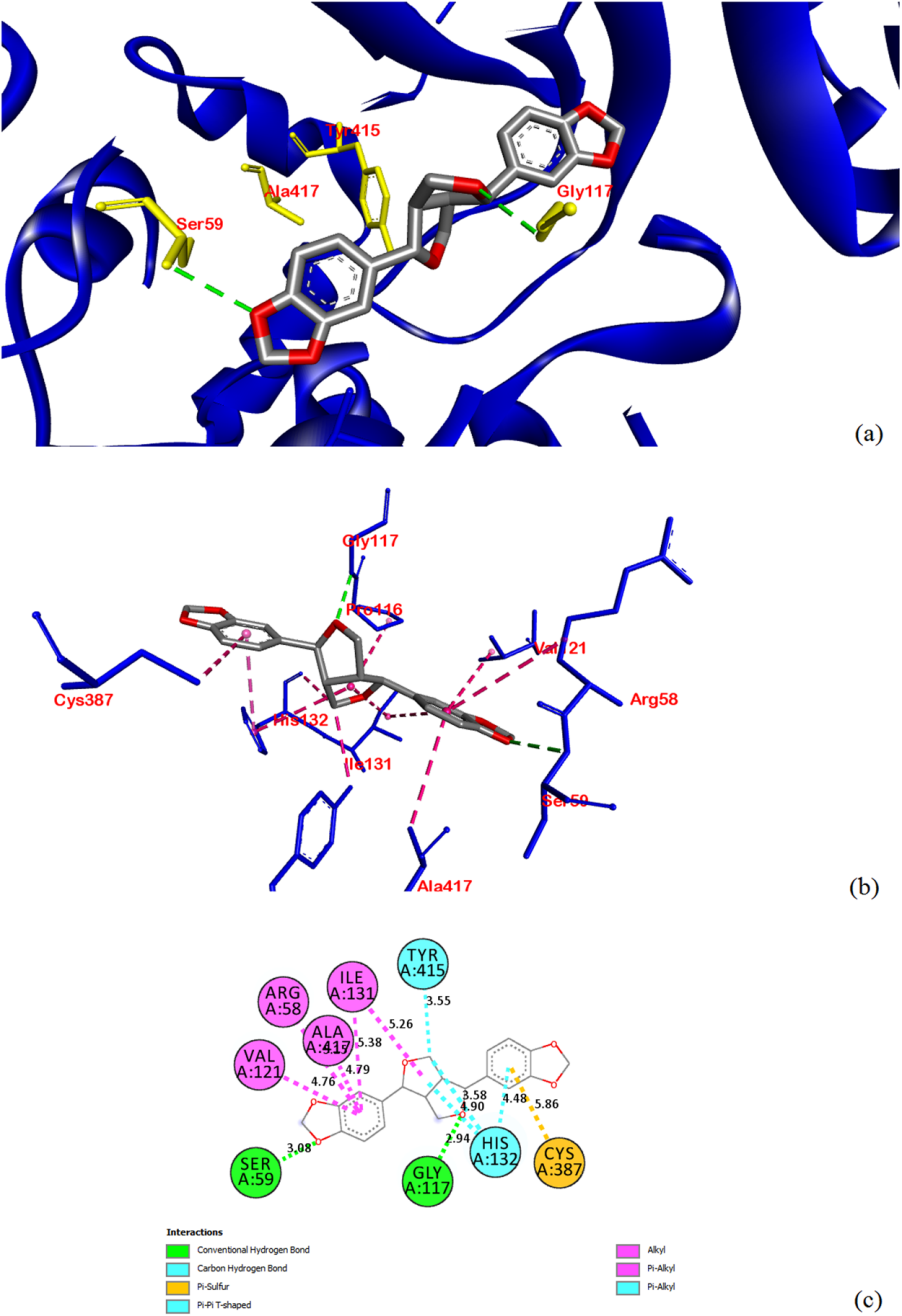
Molecular interactions between (+)-sesamin and *Mt*DprE1. Docked pose of (+)-sesamin in the *Mt*DprE1 binding site **(a)**, interactions between (+)-sesamin and *Mt*DprE1 showing key hydrogen-bonds (green dashed lines), hydrophobic bonds (dark pink dashed lines) and respective amino acid residues **(b)**, 2D plot of interactions between (+)-sesamin and key residues of *Mt*DprE1 **(c)** generated by BIOVIA Discovery Studio visualizer.

Specific interactions were further explored to understand the nature of the intermolecular bonds formed between selected compounds and the binding site residues for the four studied enzymes (Table S2). The binding poses obtained for the best binding ligands isonymphaeol D and (+)-sesamin were visually inspected and are depicted in Figs 1 and 2, respectively. We observed that isonymphaeol D showed some key molecular interactions with the key residues Pro280, Phe402 and His311 of *Mt*KasA (Fig. 1) and (+)-sesamin interacted with the key residues Cys387, Ser59 and Gly117 of *Mt*DprE1 (Fig. 2). The best score towards *Mt*PanK was observed for the flavonoid pinobanksin-3-(*E*)-caffeate (−10.0 kcal/mol) but protein-ligand interactions were not investigated for this compound as this ranked lower than the score obtained for the control inhibitor ZVT (−10.9 kcal/mol). The highest affinity towards *Mt*PknB was observed for the flavonoids pachypodol and pinobanksin-3-(*E*)-caffeate (−9.1 kcal/mol) but again this ranked lower than the score obtained for the control inhibitor mitoxantrone (−10.8 kcal/mol) and was not investigated any further.

## Discussion

In this study, we investigated the anti-TB potential of a range of propolis compounds using a guided molecular docking approach with a view to characterise their affinity towards the four mycobacterial enzymes, *Mt*PanK, *Mt*DprE1, *Mt*PknB and *Mt*KasA. The rationale for the selection of these particular proteins was that these are key enzymes required for *M*. *tuberculosis* to grow and survive within the eukaryotic host. They are also involved in a variety of essential mycobacterial pathways such as cell wall biogenesis, cofactor biosynthesis and signal transduction. They are absent in mammalian cells, which makes them highly selective and attractive ‘druggable’ targets for mycobacterial diseases, and they represent some newly-validated emerging targets against which no marketed drug is currently available^33–36^. Pantothenate kinase type I from *M*. *tuberculosis* (*Mt*PanK) is an enzyme that catalyses the first step in the biosynthesis of the cofactor Coenzyme A (CoA) by converting pantothenate (vitamin B_5_) to 4′-phosphopantothenate^33^. Serine/threonine protein kinases, such as protein kinase B (*Mt*PknB), which is implicated in the regulation of mycobacterial cell morphology, play an important role in signal transduction pathways and allow *M*. *tuberculosis* to grow and survive successfully within the host^35,42^. As the mycobacterial cell wall is a complex structure comprising layers of peptidoglycan, arabinogalactan, lipoarabinomannan and some mycolic acids, two key protein targets in the *M*. *tuberculosis* cell wall biosynthesis, β-ketoacyl acyl carrier protein synthase I (*Mt*KasA) and decaprenylphosphoryl-β-D-ribose 2′-epimerase 1 (*Mt*DprE1), were also included in this study^34,36^. The presence of mycolic acids is a unique feature of the mycobacterial cell wall. These very-long chain fatty acids, which have been linked with the ability of mycobacteria to survive in the host and to resist many antibiotics, are produced through the activity of a range of fatty acid synthases (FAS). *Mt*KasA is one of the enzymes of the mycobacterial type II FAS pathway, which is only found in bacteria^36^. A closer look at the interactions between isonymphaeol D and *Mt*KasA reveals that the prenylated tail of this flavonoid binds to the hydrophobic pocket of *Mt*KasA that contains Pro280 and Phe402. Furthermore, a strong hydrogen bond (contact distance 2.77 Å) was observed between the C-4′ phenolic oxygen of isonymphaeol D and a nitrogen of the His311 residue at the active site, in close similarity to what has been previously described as the mode of binding of the TLM control^36^. The mycobacterial cell wall enzyme decaprenylphosphoryl-β-D-ribose 2′-epimerase 1 (*Mt*DprE1) participates in the biosynthesis of two fundamental mycobacterial cell wall components, namely arabinogalactan and lipoarabinomannan^43^. The Cys387 in the active site of *Mt*DprE1 has been identified as a critical residue for the binding, through a covalent bond, of the control inhibitor 0T4 (also called CT325)^34^. In the case of (+)-sesamin, the interactions observed were not via covalent bonds but involved a π-sulfur interaction with Cys387, and strong hydrogen bonds between oxygens of the methylenedioxy and the tetrahydrofuran moieties and Ser59 and Gly117 (contact distances 3.08 and 2.94 Å, respectively).

Previous studies have reported on the anti-TB activity of some of the propolis constituents investigated here, including acacetin, apigenin, quercetin, pinostrobin, pinocembrin, naringenin, liquiritigenin, genistein, cycloartenol, β-amyrin acetate, pimaric acid, methyl caffeate, *p*-coumaric acid, methyl coumarate, cinnamic acid, medicarpin, resveratrol and sesamin^44–61^. Among the selected compounds showing the best docking scores, only β-amyrin acetate and sesamin have previously displayed moderate activity against *M*. *tuberculosis* H37Rv with minimum inhibitory concentration (MIC) values of 100 and 50 μg/mL (213 and 141 μM), respectively^53,61^. To the best of our knowledge, there have been no published reports on the antitubercular activity of isonymphaeol D. For the control inhibitors, experimental data revealed MIC values > 64 μg/mL (equivalent to >150 μM) for ZVT and 62.5 μM for thiolactomycin against *M*. *tuberculosis* H37Rv^33,62^. The activity of mitoxantrone (MIX) against *M*. *tuberculosis* H37Rv, *M*. *smegmatis mc*^2^ 155 and *M*. *aurum* A+ found MIC values in the range 25–400 μM^35^ while the activity of 0T4 was reported in terms of IC_50_ values of 10.4 and 4.6 μg/mL against *M*. *smegmatis* and *M*. *bovis* BCG, respectively^34^. In addition to this, enzymatic studies further revealed that the control compounds ZVT and MIX inhibited *Mt*PanK and *Mt*PknB with IC_50_ values of 1.13 μM and 0.8 μM, respectively^33,35^.

The purpose of molecular docking is to use scoring algorithms to estimate the likelihood that a given compound will bind to a protein target. We have identified the lignan (+)-sesamin and the prenylated flavonoid isonymphaeol D from propolis as being the best predicted binding ligands for *Mt*DprE1 and *Mt*KasA, respectively. Interestingly, isonymphaeol D displayed a strong predicted binding towards both enzymes, which suggests that it is a particularly promising agent as it has been demonstrated that the odds of successfully discovering active compounds using structure-based virtual screening methodologies are greater when a single compound can target multiple proteins^63^. Both (+)-sesamin and isonymphaeol D showed docking scores ranking higher than those obtained for the known control inhibitors of the target proteins and had predicted activities at the target sites lower than 0.1 μM. There was, however, a lack of correlation between the strong predicted affinity of (+)-sesamin for *Mt*DprE1 (score of −10.7 kcal/mol and Ki of 0.01 μM) and its observed (moderate) activity on whole bacterial cells (MIC of 141 μM). It has been previously reported that a direct correspondence between *in silico* molecular docking results and *in vitro* biological parameters cannot always be established. This can be due to the fact that some compounds are not able to go through the complex mycobacterial cell wall, or the characteristics of the binding site where inhibition takes place is different *in vivo*^64^. Isonymphaeol D and (+)-sesamin may not be used as such clinically. However, as most bioactive natural products, they represent some potentially interesting “hits”^65^ that can be further structurally optimised for the design of new anti-TB drugs and they warrant further *in vitro* biological evaluation.

## Methods

### Ligand selection

The ligands selected for this study were 78 well-characterised phytochemicals previously isolated from Algerian^66–69^, Egyptian^70–73^, Tunisian^74^, Libyan^75^, Congolese^76^, Ghanaian^77^, Kenyan^78^ and Nigerian^79^ propolis. All chemical structures were retrieved from the PubChem compound database (NCBI) (http://www.pubchem.ncbi.nlm.nih.gov).

### Ligand and protein preparation

Each ligand structure was drawn using ChemOffice v.15.1 and geometry optimised using MM2 energy minimisation^80^. All rotatable bonds present on the ligands were treated as non-rotatable. This allowed us to perform rigid docking and minimise standard errors (typically of 2.85 kcal/mol) likely due to ligands with many active rotatable bonds^81^. The Gasteiger charge calculation method was used and partial charges were added to the ligand atoms prior to docking^82^. The crystal structures of *Mt*PanK type 1 (PDB ID: 4BFT), *Mt*DprE1 (PDB ID: 4FF6), *Mt*PknB (PDB ID: 2FUM) and *Mt*KasA (PDB ID: 2WGE) were retrieved from the RCSB Protein Data Bank (PDB) database (http://www.pdb.org). The structures of the ligand inhibitors 2-chloro-N-[1-(5-{[2-(4-fluorophenoxy)ethyl] sulfanyl}-4-methyl-4h-1,2,4-triazol-3-Yl) ethyl]benzamide (ZVT) for *Mt*PanK, 3-(hydroxyamino)-N-[(1r)-1-phenylethyl]-5- (trifluoromethyl)benzamide (0T4) for *Mt*DprE1, mitoxantrone (MIX) for *Mt*PknB and thiolactomycin (TLM) for *Mt*KasA were retrieved from their corresponding PDB entries (http://www.ebi.ac.uk/thornton-srv/databases/cgi-bin/pdbsum/GetPage.pl?pdbcode=index.html). Each protein was used as a rigid structure and all water molecules and hetero-atoms were removed using BIOVIA Discovery Studio Visualizer v.4.5 (Accelrys).

### Identification of binding site residues

Previous studies were used to identify the nature and the role of the binding site residues for *Mt*PanK type 1^33^, *Mt*DprE1^34^, *Mt*PknB^35^ and *Mt*KasA^36^. Specific amino acids involved in ligand/protein interactions were also confirmed following the analyses of the PDB crystal structures available for each target protein in complex with either natural substrates or control inhibitors (Table S3).

### Grid box preparation and docking

All file conversions required for the docking study were performed using the open source chemical toolbox Open Babel v. 2.3.2^83^. Grid box parameters (Table 2) were set in such a way so as to allow for a suitably-sized cavity space large enough to accommodate each compound within the binding site of each protein and were determined using AutoDock Tools v. 1.5.6rc3^84^. Molecular docking calculations for all compounds with each of the proteins were performed using AutoDock Vina v. 1.1.2^81^. To validate the accuracy of the docking and to allow a comparison between docking scores, all co-crystallised inhibitory ligands were re-docked into the corresponding protein structures. Different orientations of the ligands were searched and ranked based on their energy scores. Our docking protocol was able to produce a similar docking pose for each control ligand with respect to its biological conformation in the co-crystallised protein-ligand complex. We further visually inspected all binding poses for a given ligand and only poses with the lowest value of RMSD (simply root-mean-square deviation) (threshold < 1.00 Å) were considered to gain a higher accuracy of docking. The Lamarckian Genetic Algorithm was used during the docking process to explore the best conformational space for each ligand with a population size of 150 individuals. The maximum numbers of generation and evaluation were set at 27,000 and 2,500,000, respectively. All other parameters were set as default. As the active binding sites and some control inhibitors for our four selected mycobacterial enzymes have been well-characterised in previous studies^33–36^, we decided to use a guided docking approach to increase docking efficiency^37^ by sampling each ligand conformation (including re-docking of the control inhibitors) in each protein binding site and then ranking these conformations using a scoring function to predict the best protein-ligand binding affinities (calculated as the predicted binding free energies ΔG_bind_ in kcal/mol) (Table S1). The lowest binding free energy (i.e. best score of the docking pose with the least root mean square deviation) indicated the highest predicted ligand/protein affinity. The Auto Dock Vina docking scores of these selected propolis constituents which ranked higher than a control inhibitor were further used to calculate the predicted inhibition constants (Ki values) of selected compounds against a given target (Table 1)^85^. Specific intermolecular interactions with the targets (Table S2 & Figs 1–2) were further visualised using BIOVIA Discovery Studio Visualizer v.4.5 (Accelrys).

**Table 2:** Grid box parameters selected for target enzymes, based on binding site residues^a^.

Target Protein (PDB ID)	Binding Site Residues-Receptor Grid Generation	Centre Grid Box (Points in X, Y, Z-axis)	Size (Points in X, Y, Z-axis)
*Mt*PanK (4BFT)	Gly 97, Ser 98, Val 99, Ala 100, Val 101, Gly 102, Lys 103, Ser 104, His 179, Tyr 235, Arg 238, Met 242, Asn 277	−18.742 × −13.919 × 11.679	20 × 20 × 20
*Mt*DprE1 (4FF6)	Gly 117, Trp 230, Val 265, Glu 336, Asn 385, Ile 386, Cys 387	14.99 × −20.507 × 37.226	20 × 20 × 20
*Mt*PknB (2FUM)	Leu 17, Gly 18, Val 25, Ala 38, Met 92, Tyr 94, Val 95, Lys 140, Met 145, Asn 143, Met 155	61.518 × 2.429 × −25.588	21 × 20 × 20
*Mt*KasA (2WGE)	Cys 171, Phe 237, Ala 279, Pro 280, His 311, Gly 318, His 345, Phe 402, Gly 403, Phe 404	38.342 × −1.206 × −7.033	20 × 20 × 20

^a^Spacing and exhaustiveness values were set up at 1 Å and 9, respectively in all cases.

## Electronic supplementary material

supplementary info

Table S1

Table S2

Table S3
