# Supplementary material for: Investigation of the anti-TB potential of selected propolis constituents using a molecular docking approach
Source: Sci Rep. 2018 Aug 16;8:12238. doi: 10.1038/s41598-018-30209-y (PMC6095843; doi:10.1038/s41598-018-30209-y)
Supplement: Supplementary file 1 — supplementary info [file 41598_2018_30209_MOESM1_ESM.docx]

**Investigation of the anti-TB potential of selected propolis constituents using a molecular docking approach**

Mohammad Tuhin Ali^1#^, Natalia Blicharska^2‡#^, Jamil A. Shilpi^3^, Veronique Seidel^4*^

^1^Department of Biochemistry and Molecular Biology, University of Dhaka, Bangladesh

^2^Victoria College, University of Toronto, Canada

^3^Pharmacy Discipline, Life Science School, Khulna University, Bangladesh

^4^Natural Products Drug Discovery Research Group, Strathclyde Institute of Pharmacy and Biomedical Sciences, University of Strathclyde, Glasgow, UK.

^‡^Current Address: Department of Pharmaceutical and Biological Chemistry, UCL School of Pharmacy, London, UK

* Corresponding author

E-mail: [veronique.seidel@strath.ac.uk](mailto:veronique.seidel@strath.ac.uk) (VS)

^#^These authors contributed equally to this work.

**Supplementary information**

**Table S1.** **Predicted binding affinity (docking scores in kcal/mol) of all propolis constituents and re-docked control inhibitors against *M. tuberculosis* target enzymes.**^a^

**Table S2. Detailed molecular interactions between selected propolis constituents and *M. tuberculosis* target enzymes.**

**Table S3: Nature and role of the binding site residues identified for each *M. tuberculosis* target enzyme.**
